# Effect of luminance noise on the object frequencies mediating letter identification

**DOI:** 10.3389/fpsyg.2014.00663

**Published:** 2014-07-03

**Authors:** Cierra Hall, Shu Wang, Reema Bhagat, J. Jason McAnany

**Affiliations:** ^1^Department of Ophthalmology and Visual Sciences, University of Illinois at ChicagoChicago, IL, USA; ^2^Department of Bioengineering, University of Illinois at Chicago, ChicagoIL, USA; ^3^Department of Electrical and Computer Engineering, University of Illinois at ChicagoChicago, IL, USA; ^4^Department of Psychology, University of Illinois at ChicagoChicago, IL, USA

**Keywords:** visual noise, letter identification, contrast sensitivity, optotype, object spatial frequency, retinal spatial frequency

## Abstract

**Purpose:** To determine if the same object frequency information mediates letter contrast threshold in the presence and absence of additive luminance noise (i.e., “noise-invariant processing”) for letters of different size.

**Methods:** Contrast thresholds for Sloan letters ranging in size from 0.9 to 1.8 log MAR were obtained from three visually normal observers under three paradigms: (1) high- and low-pass Gaussian filtered letters were presented against a uniform adapting field; (2) high- and low-pass Gaussian filtered letters were presented in additive white luminance noise; and (3) unfiltered letters were presented in high- and low-pass Gaussian filtered luminance noise. A range of high- and low-pass filter cutoffs were used to limit selectively the object frequency content of the letters (paradigms 1 and 2) or noise (paradigm 3). The object frequencies mediating letter identification under each paradigm were derived from plots of log contrast threshold vs. log filter cutoff frequency.

**Results:** The object frequency band mediating letter identification systematically shifted to higher frequencies with increasing log MAR letter size under all three paradigms. However, the relationship between object frequency and letter size depended on the paradigm under which the measurements were obtained. The largest difference in object frequency among the paradigms was observed at 1.8 log MAR, where the addition of white noise nearly doubled the center frequency of the band of object frequencies mediating letter identification, compared to measurements made in the absence of noise.

**Conclusion:** Noise can affect the object frequency band mediating letter contrast threshold, particularly for large letters, an effect that is likely due to strong masking of the low frequency letter components by low frequency noise checks. This finding indicates that noise-invariant processing cannot necessarily be assumed for large letters presented in white noise.

## INTRODUCTION

Letter optotypes are commonly used as test targets in basic studies of visual performance as well as in the clinical evaluation of visual function. An important consideration in the use of letter targets is that their Fourier spectra contain a broad range of object spatial frequencies, designated in cycles per letter (cpl; Parish and Sperling, 1991; Poder, 2003). Although visual sensitivity for spatially broad-band letter optotypes could potentially be based on any of the object frequencies contained in the letter, studies have shown that only a narrow band of object frequencies mediates contrast sensitivity (Alexander et al., 1994; Solomon and Pelli, 1994; Chung et al., 2002; Majaj et al., 2002; McAnany and Alexander, 2008; Oruc and Landy, 2009) and visual acuity (Anderson and Thibos, 1999). Furthermore, the narrow band of object frequencies that mediates performance depends on letter size, such that higher object frequencies (i.e., the edges of the letter) are used for larger letter sizes, whereas lower object frequencies are used for smaller letter sizes (Chung et al., 2002; Majaj et al., 2002; McAnany and Alexander, 2008; Oruc and Landy, 2009; Alexander and McAnany, 2010; McAnany et al., 2011).

The standard approach for studying the object frequency information mediating visual acuity and contrast sensitivity for letters has been to remove or mask selected object frequencies contained in the letter, and then measure the effect on performance. That is, if removing a range of object frequencies does not affect performance, then those frequencies must not be necessary for the task. Conversely, if removing a range of object frequencies impairs performance, then those frequencies must be useful for performing the task. Two distinct approaches based on this logic have been used to identify the object frequencies mediating letter contrast sensitivity: a letter filtering approach (Alexander et al., 1994; Chung et al., 2002; McAnany and Alexander, 2008; Alexander and McAnany, 2010) and a noise masking approach (i.e., “critical-band noise masking”; Solomon and Pelli, 1994; Majaj et al., 2002; Oruc and Landy, 2009). The former approach involves selectively removing object frequencies from the letter by spatial filtering, whereas critical-band noise masking attenuates the usefulness of selected object frequencies by masking them with spatially filtered luminance noise. Despite differences in approach, previous studies that have examined the effect of letter size on the object frequencies mediating contrast sensitivity are in good agreement. For example, the data of Chung et al. (2002), who used band-pass filtered letters, indicate that a linear function with a slope of approximately 1/3 describes the relationship between log object frequency and log letter size, for letters sizes of approximately 0.1–1.4 log MAR. Similarly, the data of Majaj et al. (2002), who used critical-band noise masking, indicate that a linear relationship with a slope of approximately 1/3 can describe the relationship between log object frequency and log letter stroke frequency, for letter sizes of approximately 0.3–2.8 log MAR.

Studies that employ visual noise as a tool to assess the object frequency information mediating contrast sensitivity or as a tool to assess visual function in patient populations (Nordmann et al., 1992; Yates et al., 1998; Levi and Klein, 2003; Pelli et al., 2004; Xu et al., 2006; Huang et al., 2007; McAnany et al., 2013) typically assume noise-invariant processing: i.e., the same mechanism and processing strategy are used in the absence and presence of noise. However, the addition of white luminance noise can alter the visual pathway mediating sensitivity under certain conditions, biasing processing from the magnocellular visual pathway towards the parvocellular visual pathway (McAnany and Alexander, 2009, 2010). Additionally, previous work has shown that higher object frequencies mediate contrast sensitivity under conditions biased toward the parvocellular pathway (McAnany and Alexander, 2008). Consequently, the addition of luminance noise might affect the object frequency band mediating letter identification. If noise affects the object frequencies mediating letter identification, then the interpretation of clinical tests that assess performance in noise (Pelli and Hoepner, 1989; Pelli et al., 2004) and basic studies of the information mediating letter identification could be complicated.

Given that the validity of noise-invariant processing in letter contrast sensitivity tasks has not been tested, the present study determined the effects of luminance noise on the object frequencies mediating contrast sensitivity for letters across a range of sizes. Estimates of object frequency were determined by low- and high-pass spatial filtering letters from the Sloan set. Letters were either presented against a uniform adapting field or in the presence of white additive luminance noise. The effects of luminance noise on object frequency were also assessed by measuring the object frequencies mediating letter identification using high- or low-pass filtered noise with the critical-band noise masking paradigm.

## MATERIALS AND METHODS

### OBSERVERS

Three of the authors (ages 22, 24, and 34 years) served as subjects. All had normal best-corrected visual acuity assessed with the ETDRS distance visual acuity chart and normal and contrast sensitivity assessed with the Pelli-Robson contrast sensitivity chart. The experiments were approved by an institutional review board at the University of Illinois at Chicago and the study adhered to the tenets of the Declaration of Helsinki.

### APPARATUS AND STIMULI

All stimuli were generated using a PC-controlled Cambridge Research Systems ViSaGe stimulus generator and were displayed on a Mitsubishi Diamond Pro (2070) CRT monitor with a screen resolution of 1024 × 768 and a 100-Hz refresh rate. The monitor, which was the only source of illumination in the room, was viewed monocularly through a phoropter with the observer’s best refractive correction. The luminance values used to generate the stimuli were determined by the ViSaGe linearized look-up table, which were verified by measurements made with a Minolta LS-110 photometer.

**FIGURE 1:**
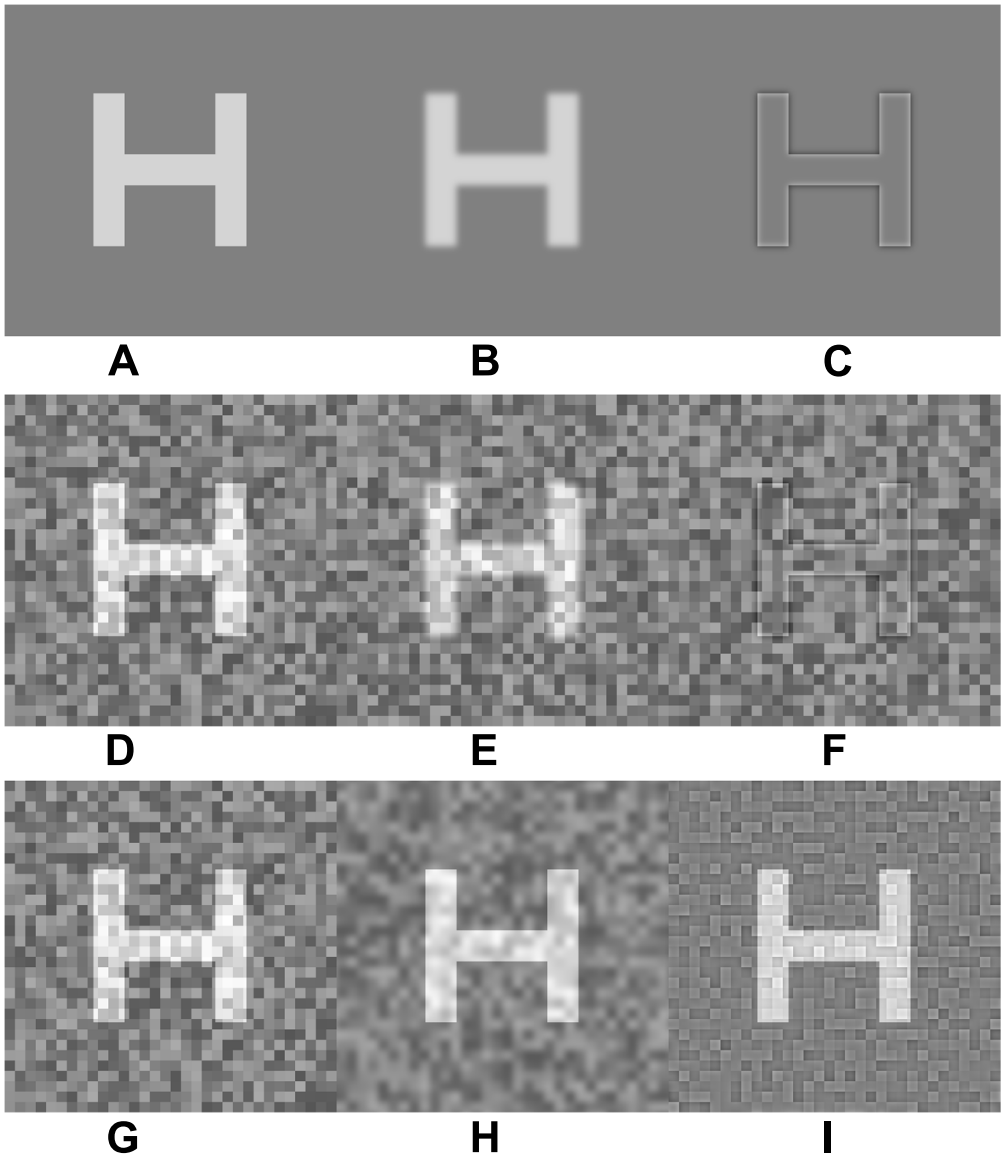
**Illustrations of the letter H presented under the three paradigms.** The top row shows examples of the H presented in the absence of noise [unfiltered H **(A)**, low-pass filtered H **(B)**, high-pass filtered H **(C)**]. The middle row shows examples of the H presented in white luminance noise [unfiltered H **(D)**, low-pass filtered H **(E)**, high-pass filtered H **(F)**]. The bottom row shows examples of the unfiltered H presented in luminance noise that was unfiltered **(G)**, low-pass filtered **(H)**, and high-pass filtered H **(I)**. The filter cutoffs were set to 7.5 cpl and the contrast of the letter was 0.66.

The test stimuli consisted of a set of ten Sloan letters (C, D, H, K, N, O, R, S, V, Z) that was constructed according to published guidelines (NAS-NRC, 1980). The Sloan letters were either unfiltered or spatially high- or low-pass filtered with a set of two-dimensional Gaussian filters. The object frequency cutoffs of the filters ranged from 0.9 to 21.0 cpl in 10 steps spaced approximately 0.15 log units apart. **Figure 1** presents examples of an unfiltered letter (**Figure 1A**), a low-pass-filtered letter (**Figure 1B**), and a high-pass-filtered letter (**Figure 1C**). The letters were of positive contrast (letter luminance higher than the adapting field luminance) and were presented at four different sizes, equivalent to 0.9, 1.2, 1.5, and 1.8 log MAR (minimum angle of resolution, where smaller values of log MAR correspond to smaller letters). This range was used in previous studies (McAnany and Alexander, 2008; Alexander and McAnany, 2010) and includes the letter size used for the Pelli-Robson contrast sensitivity chart for the standard 1 m test distance (1.5 log MAR). The letters were presented for an unlimited duration at the center of a 50 cd/m^2^ adapting field that subtended 10.7^∘^ horizontally and 8.0^∘^ vertically.

Letters were presented either in the absence of noise (**Figures 1A–C**) or in the presence of luminance noise (**Figures 1D–I**). The same letter targets shown in the absence of noise (first row) are shown in the presence of white additive luminance noise in the second row (**Figures 1D–F**). The bottom row of **Figure 1** provides examples of the stimuli used in the critical-band noise masking experiments. Examples of an unfiltered letter are shown in white noise (**Figure 1G**), in low-pass filtered noise (**Figure 1H**), and in high-pass filtered noise (**Figure 1I**). The noise field covered an area that was approximately 1.5 times larger than the letter and consisted of independently generated square checks with luminances drawn randomly from a uniform distribution with a root-mean-square (rms) contrast of 0.18. The mean luminance of the noise field was equal to that of the adapting field (50 cd/m^2^). The size of the noise checks was scaled with letter size such that there were always 15 noise checks per letter (six checks per letter cycle, as each letter contains 2.5 cycles), which maintains a constant signal-to-noise ratio (SNR) across different letter sizes. Previous work has shown that a minimum of four checks per cycle are needed to ensure that the noise is effectively white at all letter sizes (Kukkonen et al., 1995). The value of six checks per letter cycle used in the preset study is consistent with that used by others (Pelli et al., 2004). The noise spectral density ranged from 6 × 10^-6^ deg^2^ at the smallest check size to 4 × 10^-4^ deg^2^ for the largest check size. The static noise field was presented synchronously with the target for an unlimited duration, such that the onset and offset of the target and noise was simultaneous.

The contrast (C) of the letters was defined as Weber contrast:

(1) $$C=\left(L_{L}-L_{B}\right)/L_{B}\,\;\,$$

where L_L_ is the luminance of the letter and L_B_ is the background luminance. Because the contrast of complex images is difficult to define (Peli, 1990), a relative definition of contrast was used to characterize the filtered letters, as in previous studies (Chung et al., 2002; McAnany and Alexander, 2008, 2010). That is, when the contrast of the original unfiltered letter was 1.0, the filtered image was assigned a relative contrast of 1.0 without rescaling.

### PROCEDURE

A brief warning tone signaled the start of each stimulus presentation. On each trial, a single letter was selected at random from the Sloan set and presented. The observer’s task was to identify the letter verbally, which was entered by the experimenter. No feedback was given. All three observers were familiar with the Sloan set and only letters from the Sloan set were accepted as valid responses. Contrast threshold for letter identification was obtained using a 10-alternative forced-choice staircase procedure. An initial estimate of threshold was obtained by presenting a letter at a suprathreshold contrast level and then decreasing the contrast by 0.3 log units until an incorrect response was recorded. After this initial search, log contrast threshold was determined using a two-down, one-up decision rule, which provides an estimate of the 76% correct point on a psychometric function (Garcia-Perez, 1998). Each staircase continued until 16 reversals had occurred, and the mean of the last 6 reversals was taken as contrast threshold. Excluding the initial search, the staircase length was typically 35–40 trials, which produced stable measurements. In one testing session, a letter size and a paradigm (filtered letter in the absence of noise, filtered letter in the presence of noise, unfiltered letter in filtered noise) were selected pseudorandomly for testing. All cutoff object frequencies for both high-pass and low-pass filtered letters (or filtered noise) were tested in a pseudorandom order within a session.

## RESULTS

### CONTRAST THRESHOLD FOR LOW- AND HIGH-PASS FILTERED LETTERS

**FIGURE 2:**
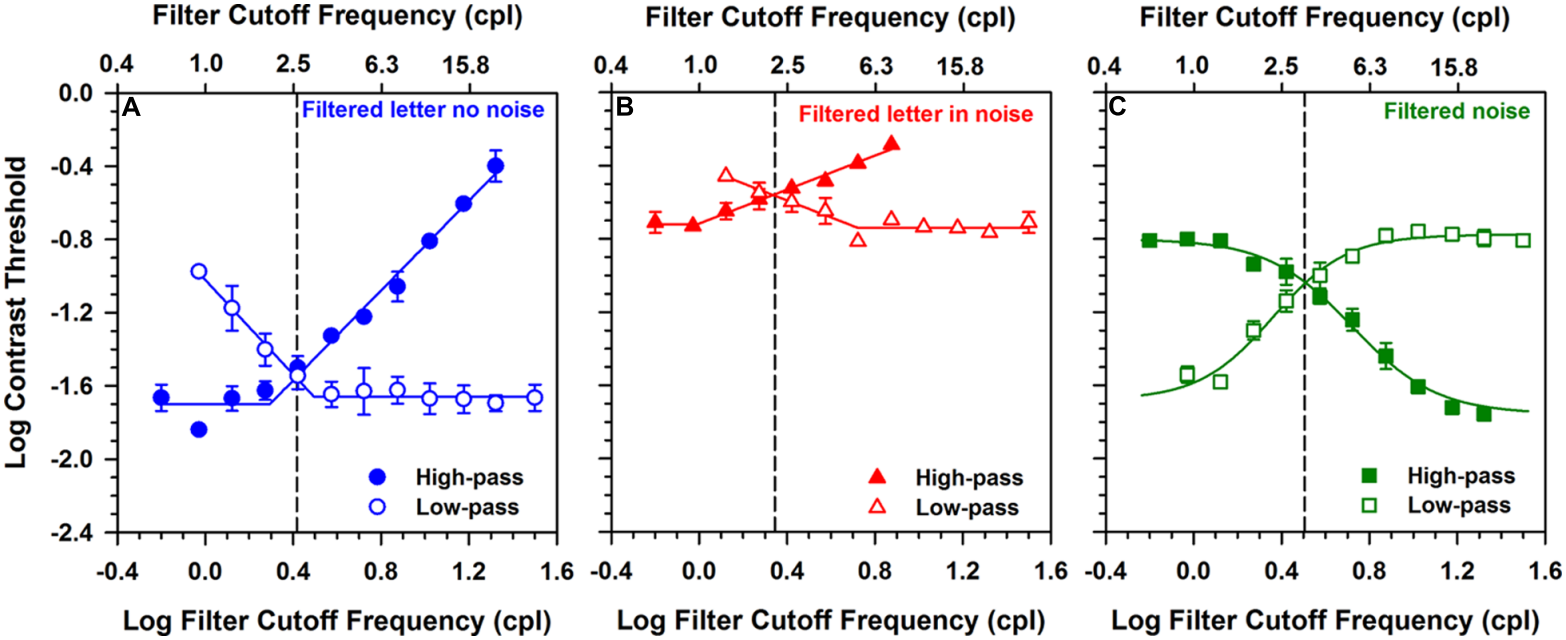
**Log contrast threshold versus log filter cutoff frequency for 1.2 log MAR letters presented in the absence of noise (A), in the presence of additive white luminance noise (B), and for unfiltered letters presented in Gaussian filtered luminance noise (C)**. Low-pass data are represented by the open symbols and high-pass data are represented by the filled symbols. Data points represent the means of three observers and error bars represent ± 1 (SEM), which are omitted when smaller than the data points. The solid lines in the left and middle panels represent piecewise linear fits to the data, whereas the solid lines in the right panel represent the function described by Majaj et al. (2002). The dashed vertical lines indicate the point at which the two functions crossed, which was used as the index of the center object frequency in the following figures.

**Figures 2A,B** show log contrast threshold for letters that were either high-pass Gaussian filtered (filled symbols) or low-pass Gaussian filtered (open symbols). These measurements were made for a letter size equivalent to 1.2 log MAR that was either presented in the absence of noise (**Figure 2A**) or in white luminance noise (**Figure 2B**). Each data point represents the mean contrast threshold value for the three subjects and the error bars are ± 1 standard error of the mean (SEM). In **Figures 2A,B**, the leftmost data points (filled circle and filled triangle, respectively) and rightmost data points (open circle and open triangle, respectively) represent contrast threshold for letters that were minimally filtered. The other data points represent the effect of successively changing the cutoff frequency of the filter to remove either the low object frequencies or the high object frequencies.

For the filtered letter data in **Figures 2A,B**, there was a region over which threshold was independent of filter cutoff and a second region over which log contrast threshold increased or decreased linearly with log filter cutoff. In order to derive the object frequency range that is used for letter identification, the data were fit piecewise with two linear functions using a least-squares criterion: one region was constrained to have a slope of 0, and the slope of the second region was unconstrained. The high-pass and low-pass functions in each plot were fit separately and are represented by the solid lines in **Figures 2A,B**. The cutoff object frequency at which the functions crossed (indicated by the vertical dashed lines) was taken as an index of the center of the object frequency region mediating letter identification. This point, which was also used in previous reports (McAnany and Alexander, 2008; Alexander and McAnany, 2010), represents approximately equal elevations of log contrast threshold, compared to the threshold values obtained with minimally filtered letters.

Log contrast thresholds for filtered letters measured in the absence of noise (**Figure 2A**) and in the presence of white noise (**Figure 2B**) differed substantially. That is, the functions measured in the presence of white noise were shifted vertically by approximately 1 log unit. This finding is expected, as high external noise levels are known to elevate contrast threshold substantially. The center object frequency was similar under both conditions (approximately 2.6 cpl in the absence of noise and 2.2 cpl in the presence of noise). Thus, the functions obtained in the absence and presence of white noise were primarily shifted vertically, with minimal horizontal shift.

The range of useful frequencies mediating contrast threshold (i.e., bandwidth) can also be derived from the plots shown in **Figures 2A,B**. The bandwidth was calculated as the full width at half-height, where half-height was obtained by averaging the minimum and crossing point thresholds. The mean bandwidth was 0.67 octaves for the letters presented in the absence of noise (**Figure 2A**) and 1.1 octaves for letters presented in white noise (**Figure 2B**). The width of the band of object frequencies has been reported to be between 1 and 3 octaves (Chung et al., 2002; Majaj et al., 2002), with minimal dependence on letter size. Thus, the bandwidth measured in white noise is within the previously reported range, whereas the bandwidth measured in the absence of noise is somewhat narrower than previous reports.

### CONTRAST THRESHOLD FOR LETTERS PRESENTED IN LOW- AND HIGH-PASS FILTERED NOISE

**Figure 2C** shows log contrast threshold for unfiltered letters presented in either high-pass Gaussian filtered (filled symbols) or low-pass Gaussian filtered (open symbols) white noise. The letter size was equivalent to 1.2 log MAR and each data point represents the mean contrast threshold value for the three subjects, with error bars representing ± 1 SEM. The leftmost point for the high-pass function (filled square) and rightmost data point for the low-pass function (open square) represent thresholds measured in noise that was minimally filtered (i.e., nearly white). The other data points represent the effect of successively changing the filter cutoff to remove object frequencies from the noise.

The data in **Figure 2C** were well fit by sigmoidal functions described previously and fit to similar data (Majaj et al., 2002; Oruc and Landy, 2009). The high-pass and low-pass functions were fit separately and are represented by the solid lines in **Figure 2C**. The crossing point of the fitted functions was taken as an index of the center of the range of frequencies mediating letter identification, to maintain consistency with the approach used in **Figures 2A,B**. Based on this definition, the center object frequency for the letters measured in filtered noise (3.2 cpl) was somewhat higher than the center frequencies in the absence of noise (2.6 cpl) or for letters in white noise (2.2 cpl). The center object frequency was also determined by calculating the derivatives of the sigmoidal curves, an approach described elsewhere (Majaj et al., 2002; Oruc and Landy, 2009). Estimates of the center object frequency based on the mean of the low- and high-pass derivatives was 3.5 cpl.

The bandwidth for letters presented in filtered noise (**Figure 2C**) was calculated using the same procedure described for the bandwidth calculations for **Figures 2A,B**. The full width at half-height of the data shown in **Figure 2C** was 2.1 octaves. This value is larger than that for filtered letters presented in the presence and absence of white noise.

### CENTER OBJECT FREQUENCY AS A FUNCTION OF LETTER SIZE

**FIGURE 3:**
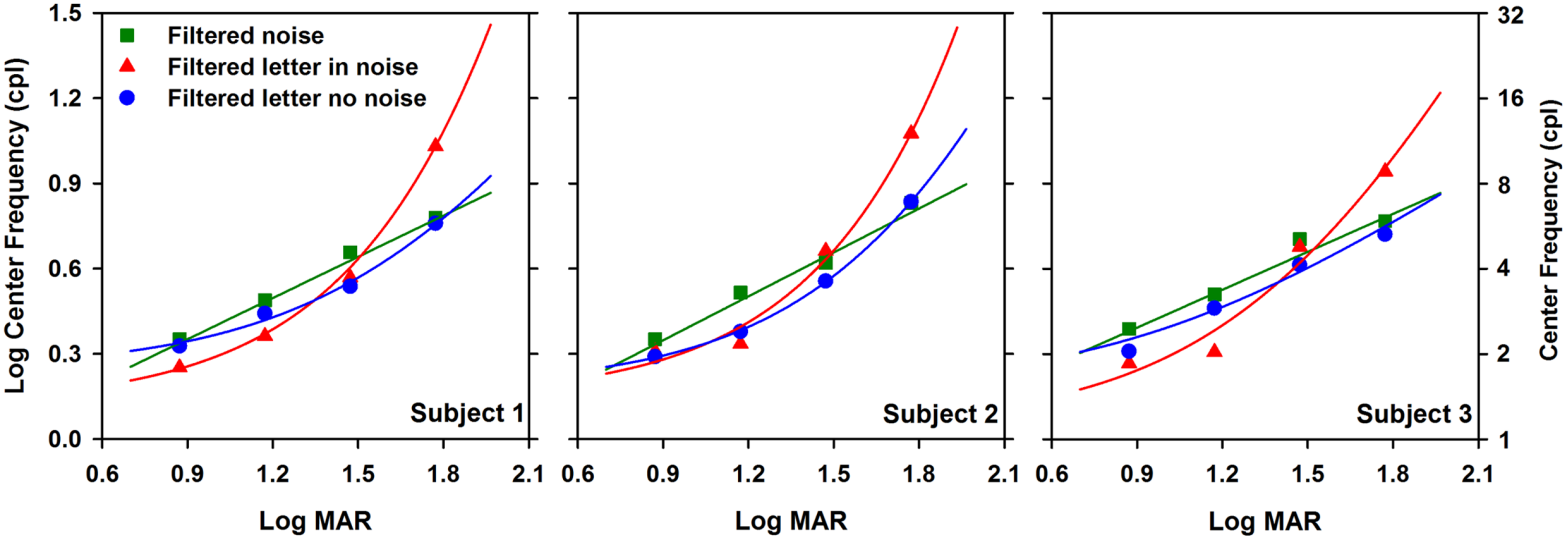
**Log center frequency (cpl) as a function of letter size (log MAR)**. Data are shown for each subject separately. Exponential curves were fit to the filtered letter in noise (triangles) and filtered letter in no noise (circles) data and a linear function was fit to the unfiltered letter in filtered noise data (squares).

The analysis illustrated in **Figure 2** was performed on the data obtained at each of the other three letter sizes, with the results shown in **Figure 3**. **Figure 3** shows log center object frequency for the three subjects as a function of log MAR letter size. The center object frequencies were based on the crossing points of the fits to the data, as described in **Figure 2**. Measurements are shown for filtered letters in the absence of noise (circles), filtered letters in the presence of white noise (triangles), and for unfiltered letters in filtered noise (squares). Data for filtered letters in the absence and presence of white noise were fit with exponential functions that transitioned from a slope of 0 for small letters to a positive slope for large letters. Data for the unfiltered letters presented in filtered noise were fit with a linear regression line, in accordance with previous reports (Majaj et al., 2002; Oruc and Landy, 2009). As can be seen from comparing the three panels, the results were highly consistent for the three subjects.

The relationship between object frequency and letter size was not identical for letters in the absence and presence of white noise. Specifically, center object frequency increased as size increased for both paradigms, but the exponential increase in center frequency for letters in white noise was greater than that for letters in the absence of noise. The largest difference between the functions measured in the presence and absence of white noise was at the largest size, where the object frequencies mediating letter identification in the presence of white noise were a factor of 1.75 higher, on average, than those measured in the absence of noise. For both paradigms, the slope of the function began to approach zero for small letter sizes, indicating that a similar constant band of frequencies mediated contrast threshold in the absence and presence of white noise for small letters. In comparison, log object frequency increased linearly with log letter size for the measurements made in filtered noise. The object frequencies mediating letter identification in filtered noise tended to be slightly, but systematically, higher than those measured in the absence of noise.

A two-way repeated measures analysis of variance (ANOVA) was performed to compare the object frequencies measured under the three paradigms at the four letter sizes. The ANOVA indicated significant main effects of paradigm [*F*(2,12) = 9.7, *p* < 0.05] and letter size [*F*(3,12) = 125.4, *p* < 0.05]. Additionally, there was a significant interaction between paradigm and size [*F*(6,12) = 32.8, *p* < 0.05]. Bonferrioni corrected *post hoc* comparisons indicated that for the 1.8 log MAR letter size, object frequency was significantly greater (*p* < 0.05) for measurements in white noise (triangles) compared to those measured in both filtered noise (squares) and in the absence of noise (circles). The *post hoc* comparisons also indicated a significant difference (*p* < 0.05) between the object frequency measured in filtered noise (squares) and that measured in white noise (triangles) at the 1.2 log MAR letter size.

**FIGURE 4:**
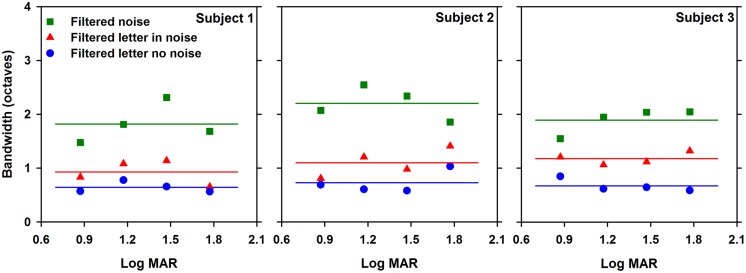
**Bandwidth (octaves) as a function of log MAR letter size**. Data are shown for each subject individually. Linear regression lines with slopes of zero are fit to the data, as described in the text.

The bandwidth of useful object frequencies mediating letter contrast threshold was also assessed for each paradigm at each letter size, with the results for each subject shown in **Figure 4**. As in **Figure 3**, measurements are shown for letters in the absence of noise (circles), letters in the presence of white noise (triangles), and for letters in filtered noise (squares). The bandwidths for each paradigm were defined as the full width at half-max, as described above. The data were fit with linear regression lines of zero slope, since there was no effect of letter size on bandwidth, as discussed below. A two-way repeated measures ANOVA was performed to compare the bandwidths measured under the three paradigms at the four letter sizes. The ANOVA indicated significant main effects of paradigm [*F*(2,12) = 106.2, *p* < 0.05], but not letter size [*F*(3,12) = 1.3, *p* > 0.05]. Of note, the finding that bandwidth is approximately independent of letter size has been reported previously (Chung et al., 2002). Additionally, there was no significant interaction between paradigm and size [*F*(6,12) = 1.5, *p* > 0.05). The estimates of mean center frequency and bandwidth (±SEM) are listed in **Table 1** for each paradigm and letter size. Additionally, **Table 1** lists the mean (±SEM) contrast threshold for unfiltered letters in the absence and presence of noise and for unfiltered letters in white noise.

**Table 1:** Center frequency, bandwidth, and contrast threshold for each letter size and paradigm.

Paradigm	Size (log MAR)	Mean center frequency ± SEM (cpl)	Mean bandwidth ± SEM (octaves)	Log contrast threshold ± SEM (unfiltered letter/noise)
Filtered letter no noise	0.9	2.04 ± 0.05	0.70 ± 0.08	-1.64 ± 0.03
	1.2	2.61 ± 0.15	0.67 ± 0.06	-1.66 ± 0.07
	1.5	3.72 ± 0.20	0.63 ± 0.02	-1.89 ± 0.08
	1.8	5.95 ± 0.47	0.73 ± 0.15	-1.73 ± 0.02

Filtered letter in noise	0.9	1.89 ± 0.07	0.95 ± 0.13	-0.82 ± 0.07
	1.2	2.21 ± 0.08	1.12 ± 0.05	-0.71 ± 0.06
	1.5	4.35 ± 0.33	1.08 ± 0.05	-0.88 ± 0.03
	1.8	10.46 ± 0.92	1.13 ± 0.24	-0.95 ± 0.01

Filtered noise	0.9	2.31 ± 0.07	1.70 ± 0.19	-0.86 ± 0.01
	1.2	3.20 ± 0.06	2.10 ± 0.23	-0.81 ± 0.02
	1.5	4.60 ± 0.26	2.23 ± 0.10	-0.88 ± 0.08
	1.8	6.22 ± 0.29	1.86 ± 0.10	-1.08 ± 0.05

**FIGURE 5:**
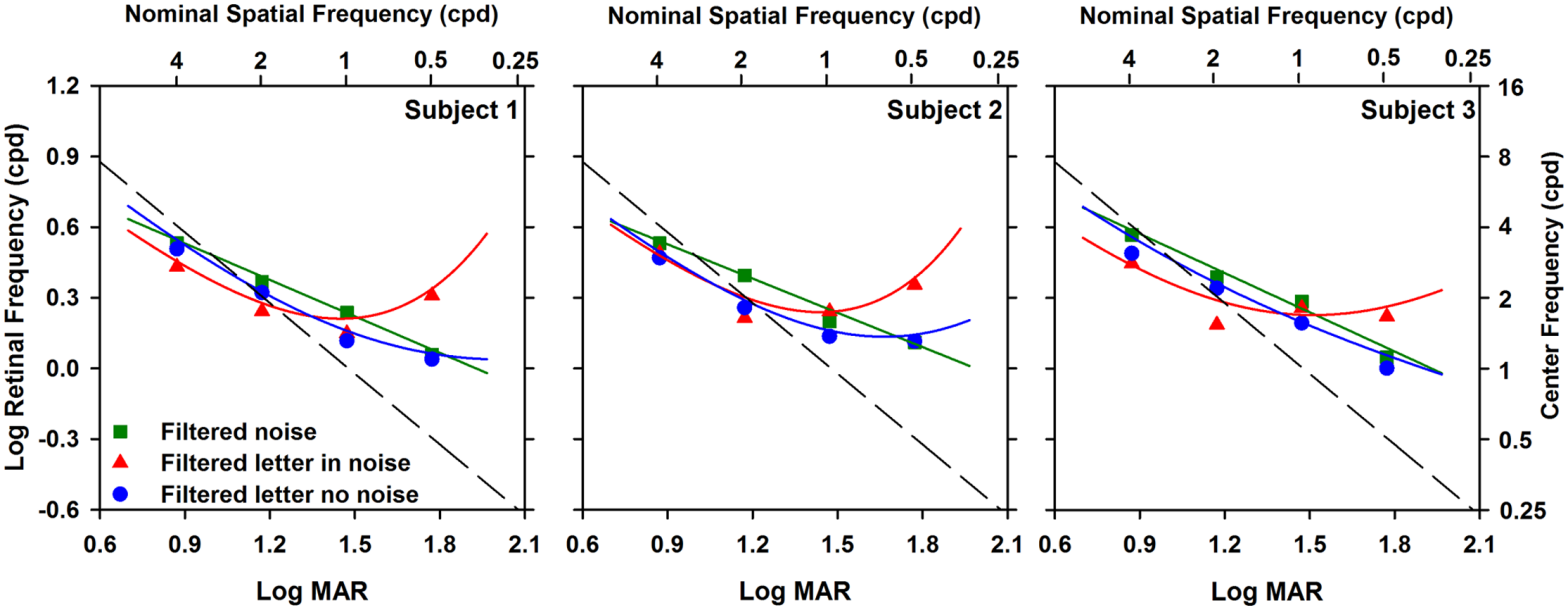
**Log retinal frequency (cycles per degree) as a function of log MAR letter size**. Data are shown for each subject individually. The data and fits are replotted from **Figure 3** in terms of retinal frequency, as described in the text. The dashed line represents the standard assumption that 30 cpd is equivalent to 0 log MAR.

The relationship between the retinal spatial frequencies (cycles per degree; cpd) mediating letter identification and log MAR letter size is shown in **Figure 5**, which replots the data and best-fit curves of each subject shown in **Figure 3** in terms of cpd. This conversion is based on the following relationship (Alexander and McAnany, 2010):

(2) $$F_{r}=\frac{12*F_{0}}{MAR}\,\;$$

where *F_r_* is retinal frequency in cpd, *F_o_* is object frequency in cpl, and *MAR* is 1/5 of the letter size in arcmin.

The top *x*-axis in **Figure 5** indicates the nominal retinal frequencies corresponding to the log MAR values. This correspondence is based on the convention that 0 log MAR (20/20 Snellen equivalent) is equivalent to a retinal frequency of 30 cpd (Regan et al., 1981). This relationship assumes that an object frequency of 2.5 cpl (equivalent to the letter stroke width) governs performance at all sizes. The diagonal dashed line in **Figure 5** represents a one-to-one relationship between the derived center retinal frequency and the nominal retinal frequency, based on this assumption. If letter identification is governed by an object frequency range centered at 2.5 cpl for all sizes, then the nominal retinal frequency would be proportional to log MAR and the data would fall along the dashed line. It is apparent that none of the curves in **Figure 5** follow the dashed line. Rather, the data points for the two smallest letter sizes tested (highest frequencies) fall near the dashed line, whereas the data points for the two largest letter sizes tested fall above the line. There is a divergence for the filtered letter in white noise function for the largest letter size (equivalent to 0.5 cpd), where the retinal frequency is slightly higher than the values at 1.0 and 2.0 cpd.

## DISCUSSION

The purpose of the present study was to determine the effects of additive luminance noise on the object frequencies mediating letter contrast threshold across a range of letter sizes. The object frequency information mediating letter contrast threshold was assessed under three paradigms: (1) letters presented against a uniform adapting field; (2) letters presented in white luminance noise; (3) letters presented in filtered luminance noise (critical-band noise masking). There were similarities among the three paradigms in that the band of object frequencies mediating contrast threshold systematically increased with increasing letter size, consistent with previous reports (Alexander et al., 1994; Solomon and Pelli, 1994; Chung et al., 2002; Majaj et al., 2002; McAnany and Alexander, 2008; Oruc and Landy, 2009). However, the functions relating log object frequency to log MAR obtained under the three paradigms had three different shapes: For letters presented in white noise, the increase in log object frequency with increasing log MAR was strongly non-linear (exponential increase); For letters presented against a uniform field, the increase in log object frequency as log MAR increased was weakly non-linear; For letters presented in filtered noise, the increase in log object frequency with increasing log MAR was linear.

Despite the differences in the shapes of the functions relating object frequency and letter size, the absolute values of object frequency were approximately similar for letter sizes ranging from 0.9 to 1.5 log MAR. If the visual pathway mediating contrast threshold had changed from the magnocellular (MC) pathway to the parvocellular (PC) pathway due to the addition of noise, as proposed as a possibility in the Introduction, an increase in object frequency would be expected for letters presented in white noise. This was not observed. The explanation for why the object frequencies did not increase for small to medium sized letters due to the addition of noise is that the PC pathway likely mediated contrast threshold under all conditions. This explanation is based on the values of object frequency obtained previously under conditions biased toward the PC pathway (Alexander and McAnany, 2010), which closely match the values obtained in the presence and absence of noise in the present study. Additional work is needed to determine how the object frequency results of the present study would differ if measured under conditions that targeted the MC pathway.

A substantial increase in object frequency was observed for the largest letter size tested (equivalent to 1.8 log MAR) in white noise, compared to the object frequencies used in the absence of noise or for letters in filtered noise. An increase in center object frequency due to the addition of white noise is also apparent in the data of Oruc and Landy (2009), but the consistency and significance of the change in their data is difficult to evaluate, as their focus was on determining whether object frequency depends on letter size in the presence of white noise. Oruc and Landy (2009) used critical-band noise masking to derive the object frequencies mediating letter identification for letters in the presence and absence of a white noise field. Despite differences in methodology, our results support their finding that the object frequencies mediating letter contrast threshold depend on letter size regardless of whether the letters are presented against a uniform field or in the presence of white noise.

A model has been proposed by Chung et al. (2002) to account for the shift in object frequency with letter size. This model suggests that the center frequency of the band of object frequencies mediating letter contrast threshold is jointly dependent on the letter sensitivity function (LSF; the object frequencies available in the letter) and the contrast sensitivity function (CSF; the relationship between contrast sensitivity and retinal frequency). This model accounts well for the change in object frequency with changes in letter size for letters of relatively small angular subtense (Chung et al., 2002). However, this model cannot account for the observed changes in center frequency for the large letter sizes used in the present study because the LSF and contrast sensitivity are both independent of letter size for letters greater than approximately 1.2 log MAR (McAnany and Alexander, 2006). Consequently, the model of Chung et al. (2002) would predict that center frequency is constant for large letters. Oruc and Landy (2009), who used white noise to flatten the CSF rather than using large letters, also reported that the center frequency changed with letter size.

At least two possible explanations could account for the large shift in object frequency due to the addition of white noise for the 1.8 log MAR letter size. First, the noise fields in the present study were designed according to previous guidelines to be effectively white (Kukkonen et al., 1995), but the substantial power in the low frequency range for large check sizes may provide enhanced masking of the low object frequencies contained in the letters. Attenuation of the low object frequencies would force the observer to use higher object frequencies (i.e., the edges of the letters), accounting for the differences observed in the presence and absence of noise. Additional work with noise fields that have a constant check size for letters of different size is needed to evaluate this explanation. As an alternative explanation, the subjects may have employed a different strategy to perform the task in the presence of noise. Previous work has indicated that the addition of noise can affect the processing strategy used to perform the task (Allard and Cavanagh, 2011). Given that the differences in object frequency among the paradigms were not the same at all letter sizes, the effects of white noise are more likely attributable to strong masking of low object frequencies contained in the large letters, rather than a shift in processing strategy. However, additional work is needed to confirm this explanation.

In addition to the differences in center object frequency among the paradigms, there were also significant differences in bandwidth among the three paradigms. Bandwidth, averaged across subjects and letter size, was slightly greater in the presence of white noise (1.1 octaves) compare to measurements made in the absence of noise (0.7 octaves). The increase in bandwidth for letters in white noise tended to be similar for all letter sizes. Thus, for the largest letter tested, the center frequency of the band of object frequencies mediating letter contrast sensitivity shifted to higher frequencies but did not become broader. The bandwidth measured for letters in filtered noise (2.0 octaves, on average), is similar to that reported previously (Solomon and Pelli, 1994; Chung et al., 2002; Majaj et al., 2002). Channel switching or “off-frequency looking” would be expected to broaden the estimated bandwidth and may provide an explanation for the larger range of frequencies used in filtered noise. For example, in the presence of low-pass filtered noise, the subject could potentially use a channel with a higher peak frequency to avoid the low-pass noise (the opposite could occur in high-pass filtered noise). Previous results indicate that under some conditions, subjects do switch channels to improve the signal to noise ratio (Oruc and Landy, 2009).

In summary, we show that the addition of noise can affect the object frequency information mediating letter identification, particularly for large letters. For letters equivalent to 1.8 log MAR, the addition of noise had marked effects on the object frequency information mediating letter identification. This finding suggests that moderate to small letter sizes may be most appropriate for comparing letter contrast threshold in the presence and absence of noise because the assumption of noise-invariant processing largely holds.

## Conflict of Interest Statement

The authors declare that the research was conducted in the absence of any commercial or financial relationships that could be construed as a potential conflict of interest.
